# Effects of Daily Melatonin Supplementation on Visual Loss, Circadian Rhythms, and Hepatic Oxidative Damage in a Rodent Model of Retinitis Pigmentosa

**DOI:** 10.3390/antiox10111853

**Published:** 2021-11-22

**Authors:** Lorena Fuentes-Broto, Lorena Perdices, Francisco Segura, Elvira Orduna-Hospital, Gema Insa-Sánchez, Ana I. Sánchez-Cano, Nicolás Cuenca, Isabel Pinilla

**Affiliations:** 1 Department of Pharmacology, Physiology & Legal and Forensic Medicine, Universidad de Zaragoza, 50009 Zaragoza, Spain; lfuentes@unizar.es (L.F.-B.); gis@unizar.es (G.I.-S.); 2 Aragon Institute for Health Research (IIS Aragón), 50009 Zaragoza, Spain; lperdices@gmail.com (L.P.); psegura@unizar.es (F.S.); eordunahospital@unizar.es (E.O.-H.); anaisa@unizar.es (A.I.S.-C.); 3 Department of Surgery, Universidad de Zaragoza, 50009 Zaragoza, Spain; 4 Department of Applied Physics, Universidad de Zaragoza, 50009 Zaragoza, Spain; 5 Department of Physiology, Genetics and Microbiology, University of Alicante, 03690 San Vicente del Raspeig, Spain; cuenca@ua.es; 6 Department of Ophthalmology, Lozano Blesa University Hospital, 50009 Zaragoza, Spain

**Keywords:** retinitis pigmentosa, antioxidant, melatonin, oxidative stress, neurodegeneration, circadian rhythms, vision, retina

## Abstract

Retinitis pigmentosa (RP) is a group of inherited neurodegenerative diseases characterized by a progressive loss of visual function that primarily affect photoreceptors, resulting in the complete disorganization and remodeling of the retina. Progression of the disease is enhanced by increased oxidative stress in the retina, aqueous humor, plasma, and liver of RP animal models and patients. Melatonin has beneficial effects against age-related macular degeneration, glaucoma, and diabetic retinopathy, in which oxidative stress plays a key role. In the present study, we used the P23HxLE rat as an animal model of RP. Melatonin treatment (10 mg/kg b.w. daily in drinking water for 6 months) improved the parameters of visual function and decreased the rate of desynchronization of the circadian rhythm, both in P23HxLE and wild-type rats. Melatonin reduced oxidative stress and increased antioxidant defenses in P23HxLE animals. In wild-type animals, melatonin did not modify any of the oxidative stress markers analyzed and reduced the levels of total antioxidant defenses. Treatment with melatonin improved visual function, circadian synchronization, and hepatic oxidative stress in P23HxLE rats, an RP model, and had beneficial effects against age-related visual damage in wild-type rats.

## 1. Introduction

Retinitis pigmentosa (RP, MIM #268000) is a group of inherited neurodegenerative diseases characterized by a progressive loss of visual function that affects not only the photoreceptors but also other retinal layers, resulting in the complete disorganization and remodeling of the retina [1]. In the early stages, individuals with this disease present with night blindness and loss of the peripheral visual field, but the consequent degeneration of the cones following the death of the rods results in the loss of central vision and complete blindness. Regardless of the primary cause, progression of the disease is enhanced by increased oxidative stress that occurs in the retina, inducing inflammation and apoptosis, and damaging macromolecules such as DNA, proteins, and lipids [2]. In addition, increased oxidative damage is observed in the aqueous humor, retina, and plasma of RP animal models and patients [3]. Not only the retina is affected; the hepatic oxidative status is also affected by free radicals in the P23H rat model of RP [4].

Melatonin (*N*-acetyl-5-methoxytryptamine) is an indolamine derived from an essential amino acid, tryptophan, and is synthesized by numerous organs, including the pineal gland, retina, thyroid, lacrimal glands, gastrointestinal tract, testicles, skin, kidneys, pancreas, spleen, erythrocytes, platelets, thymus, placenta, Harderian glands, and others [5]. This molecule is found not only in mammals, but also in other vertebrate as well as invertebrate animals, unicellular organisms, bacteria, and plants [6]. Due to its presence in edible plants and foods, including milk, fruits, vegetables, and fish, and its high content in some medicinal herbs, including chamomile and St John’s wort, melatonin is used to treat some diseases in traditional Chinese medicine [7,8].

Although melatonin is mainly known for its role in regulating circadian rhythms, it is involved in various physiological functions, such as cardiovascular and immune system regulation, retinal function, and the synchronization of peripheral oscillators (peripheral tissues such as the heart and pancreas) [9]. Melatonin is the focus of this study due to its well-known antioxidant effects, scavenging free radicals, and protective effects against oxidative stress [10]. Melatonin has been proposed as a therapeutic and neuroprotective agent in neurodegenerative and age-related diseases [11], in which oxidative damage is an important factor implicated in their pathogenesis and progression.

When melatonin is produced by the retina, it appears to have a local effect within the retina itself, in addition to acting as a neuromodulator of retinal function. This would influence retinomotor responses, modulating neurotransmitter release (dopamine release), the phagocytosis of rod outer segment discs, and light sensitivity [12,13].

In relation to degeneration, melatonin increases autophagy in favor of cell survival [14,15,16]. It is also a powerful antioxidant, as it interacts directly with free radicals and neutralizes them [17]. Likewise, its antiapoptotic efficacy in the retinal pigment epithelium has been demonstrated [18,19]. Melatonin decreases the concentration of synaptic glutamate in glaucoma, and therefore provides neuroprotection against excitotoxic damage [20]. It also acts on vascular endothelial growth factor secretion and cell migration [21], and stimulates telomerase activity in age-related macular degeneration (DMAE) [22].

The aim of this study was to analyze the neuroprotective effects of melatonin against retinal degeneration and hepatic oxidative damage in the P23H rat, an animal model of RP.

## 2. Materials and Methods

### 2.1. Animals and Treatments

P23H line 1 homozygous albino rats, an animal model of RP, were obtained from Dr. Matthew LaVail (USCF). P23H rats were crossed with wild-type Long–Evans (LE) rats (Charles River Laboratories, Barcelona, Spain) to generate transgenic-pigmented offspring (P23HxLE). As a reference group, Sprague Dawley (SD) rats were also crossed with LE rats to produce pigmented and healthy animals (SDxLE). The animals were bred in a colony and maintained in the animal facilities of the Aragon Institute for Health Research (IIS Aragón) under controlled humidity (60%), temperature (23 ± 1 °C), and photoperiod (LD 12:12) conditions. All procedures were carried out according to the Spanish Policy for Animal Protection RD53/2013, which meets the guidelines for the ethical use of animals from the European Council (Directive 2010/63/EU) and approved by the Ethics Committee for Clinical Research of Aragon from the University of Zaragoza (project license PI12/14).

P23HxLE and SDxLE animals were divided into 2 groups, with five animals per group: group 1, without antioxidant treatment in the drinking water (sham group), and group 2, with melatonin (MT) dissolved in the drinking water (MT group) for 6 months.

Melatonin (Fagron Ibérica, 33457-24, Terrassa, Spain) solution was prepared freshly twice per week by first being dissolving in a minimal amount of ethanol (0.2%) due to its poor water solubility, and then in the drinking water at a concentration of 10 mg/kg b.w. per day. To avoid variations in results because of the addition of the ethanol, groups without treatment were also provided the same percentage of ethanol in the drinking water. To reduce the oxidation process of melatonin, the water bottles were light-sealed throughout the experiment.

### 2.2. Visual Assessment: Visual Acuity and Contrast Sensitivity

Assessment of visual function was performed using an OptoMotry system (OptoMotry™, CerebralMechanics, Lethbridge, AB, Canada) [23]. This system was used to evaluate the visual acuity (VA) and contrast sensitivity (CS) of both P23HxLE and wild-type rats monthly from postnatal day 30 (P30) to P180. The maximum VA and minimum contrast identified were reached when the rat could not distinguish the stimuli presented and were judged by the experimenter using a video camera, which provides real-time feedback on a computer. VA and CS results depend on the experience and training of the experimenter, and thus all the rat-tracking movements were assessed by the same researcher.

### 2.3. Electroretinogram Recordings

After 8 h of dark adaptation, animals were anesthetized with ketamine and xylazine (90/10 mg/kg b.w. i.p) in dim red light. Recordings were assessed at P180 following a topical drop of 1% tropicamide (Colircusí Tropicamida; Alcon Labs, Barcelona, Spain) to dilate the pupils in addition to saline solution and a drop of 2% Methocel (OmniVision, Puchheim, Germany) to allow for proper electrical contact between the cornea and electrodes (gold wire loop). A heating pad was used to maintain a stable temperature (37 °C) during the procedure. An Espion system from Diagnosys LLC (Cambridge, UK) presented the stimulus and acquired the data following a previously described protocol [24].

#### 2.3.1. Mixed b-Wave

To measure the contributions of the rod and cone pathways, dark b-wave amplitudes were studied. Rats were presented with 3 to 8 single flashes of 10 µs duration, and responses recorded. As stimuli, 10 intensities increasing from −3.70 to 2.86 log cd·s/m^2^ were presented. To reduce the consequences of photopigment bleaching, interstimulus intervals (ISIs) started at 10 s with the lowest intensity (−3.70 log cd·s/m^2^), increasing until 120 s at the highest intensity (2.86 log cd·s/m^2^).

#### 2.3.2. Double-Flash Protocol

After 10 min of light adaptation, the double-flash protocol was carried out to obtain isolated cone and rod responses, according to a previously described protocol [25] where the first, conditioning flash activates both rods and cones. With the interstimulus interval being short enough, the rods are still saturated by the first flash, making them unresponsive to the second, probe flash; therefore, with the probe flash we obtain the isolated cone function. The isolated rod function is obtained by subtracting the cone function from the initial, mixed function in response to the conditioning flash.

### 2.4. Telemetry: Body Temperature and Locomotor Activity Recording

To assess the circadian rhythm of P23HxLE and wild-type rats, the activity and core temperature were measured through an intraperitoneally implanted transmitter (TA-T20^®^, Data Sciences International, St. Paul, MN, USA). Rats were separated into individual cages and connected to a receiver that collects activity and temperature data every 10 min for 1 week using Dataquest A.R.T software (Data Sciences International, New Brighton, MN, USA). El Temps^®^ software (1.292, Díez Noguera, University of Barcelona, Barcelona, Spain) was used to obtain a cosinor analysis (including mesor, amplitude, acrophase, and period), actograms, mean waveforms, and periodograms [26]. In addition, Circadianware^®^ (University of Murcia, Murcia, Spain) was used for a non-parametric analysis of the data [27].

### 2.5. Sample Collection and Preparation

Animals were killed by carbon dioxide asphyxiation when the treatment period ended (6 months). Whole liver tissue was removed and quickly homogenized, as described previously [28], in a 0.2 M phosphate buffer composed of Na_2_HPO_4_ (Panreac, A1046, Barcelona, Spain NaH_2_PO_4_ (Panreac, A3559, Barcelona, Spain) (pH = 7.4), 0.5% Triton X-100 (Panreac, A4975, Barcelona, Spain), 5 mM of β-mercaptoethanol (M6250, Sigma Aldrich, Madrid, Spain), and 0.1 mg/mL phenylmethylsulfonyl fluoride (P7626, Sigma Aldrich, Madrid, Spain). Aliquots were frozen at −80 °C for biochemical analyses.

### 2.6. Measurement of Oxidative Stress Parameters

The total protein present in the liver tissue was quantified by the Bradford method [29] to normalize the protein concentrations.

Hepatic lipid peroxidation was determined by the concentration of malondialdehyde and 4-hydroxyalkenal. Protein carbonyl groups were calculated as a marker of oxidized proteins and nitrite levels as an indicator of nitrosative damage. The ratio of reduced glutathione (GSH) to oxidized GSH (GSSG) is a useful indicator of cellular health. All protocols were previously optimized [4]. In brief, MDA and 4-HDA were measured by their reaction with *N*-methyl-2-phenylindole at 45 °C, forming a colorimetric product with maximal absorbance at 586 nm [30]. Carbonyl groups were measured when reacting with 2,4-dinitrophenylhydrazine (D199303, Sigma Aldrich, Madrid, Spain), resulting in stable 2,4-dinitrophenylhydrazone products, UV–Vis spectrophotometrically at 375 nm [31]. Nitrites react with the Griess reagent (03553, Sigma Aldrich, Madrid, Spain), turning into a pink/purple product that is measured spectrophotometrically at 550 nm [32]. Sulfhydryl groups (GSH) react with Ellman’s reagent (5,5′-dithiobis-2-nitrobenzoic acid (DNTB) (D8130, Sigma Aldrich, Madrid, Spain), resulting in a yellow product (5-thio-2-nitrobenzoic acid) that can be measured at 412 nm [33]. 4-vinylpyridine (L13316AC, AlfaAesar Thermo Fisher Scientific, Madrid, Spain) prevents GSH reacting to DTNB, making a pyridinium salt [34] and allowing the GSH/GSSG ratio to be measured.

### 2.7. Determination of Antioxidant Status

The measurement of antioxidant defenses was determined by total antioxidant capacity and superoxide dismutase (SOD), antioxidant activities of catalase (CAT), and glutathione S-transferase (GST) using previously described protocols [4]. In brief, total antioxidant capacity was measured as the inverse of the amount of ABTS radical (ABTS^•+^) formed when 2,2′-azino-bis(3-ethylbenz-thiazoline-6-sulfonic acid (ABTS; A1888, Sigma-Aldrich, Madrid, Spain) is oxidized by hydrogen peroxide (H1009; Sigma Aldrich, Madrid, Spain) and metmyoglobin (M1882, Sigma-Aldrich, Madrid, Spain) [35]. CAT (CAT, EC 1.11.1.6) activity was measured following the decrease in H_2_O_2_ for 30 s at 240 nm. SOD (SOD, EC 1.15.1.1) activity was based on the inhibition of the rate of reduction in cytochrome c (C2506, Sigma-Aldrich, Madrid, Spain) by O_2_^•−^, with a xanthine (X7375, Sigma-Aldrich, Madrid, Spain)/xanthine oxidase (X4500, Sigma-Aldrich, Madrid, Spain) system as a source of O_2_^•−^. GST (GST, EC 2.5.1.18) catalyzes the reaction of the sulfhydryl groups of the GSH with 1-chloro-2,4-dinitrobenzene (CDNB) (237329, Sigma Aldrich, Madrid, Spain). The result is a conjugate GSH-CDNB, which can be detected by spectrophotometry at 340 nm.

### 2.8. Statistical Analysis

Statistical analyses were performed with IBM SPSS Statistics 26 (IBM Corp, Armonk, NY, USA), and graphs were made with GraphPad Prism version 8 (GraphPad Software, San Diego, CA, USA).

For each group, the mean ± standard error of the mean was plotted. To test for significant differences among groups, a non-parametric test, the Kruskal–Wallis test, was used, with the subsequent comparison between the groups carried out by using the Mann–Whitney U test. *p* values < 0.05 were considered significant for all hypotheses tested.

## 3. Results

### 3.1. Visual Parameters

**Figure 1:**
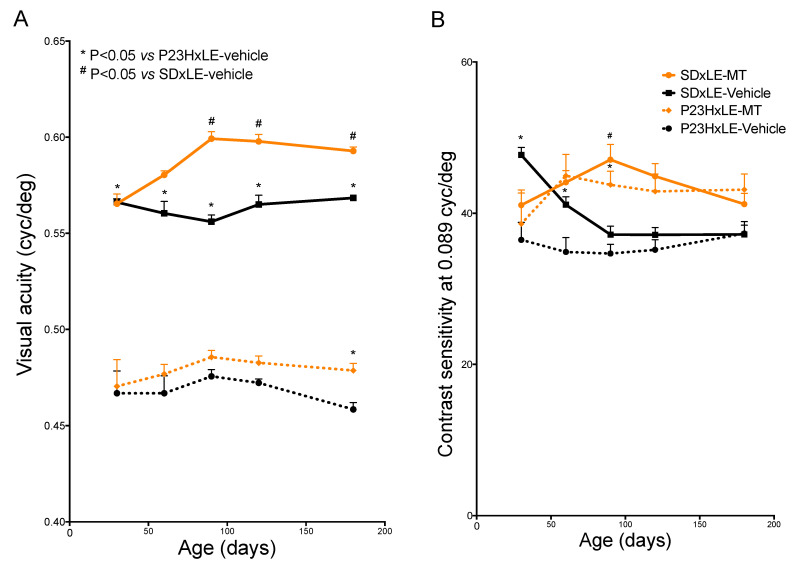
Visual acuity (VA) (**A**) and contrast sensitivity (CS) (**B**) values obtained in different treatment groups in P23HxLE and SDxLE rats: P23HxLE-vehicle, P23HxLE-MT, SDxLE-vehicle, and SDxLE-MT. Data obtained at P30, 60, 90, 120, and 180 by optometry on average between the anti- and clockwise directions. Data plots show the mean ± SEM (*n* = 5/group). Mann–Whitney U test: # *p* < 0.05 versus SDxLE animals, * *p* < 0.05 versus P23HxLE-vehicle animals. MT, melatonin.

Both VA and SC parameters showed lower values in P23HxLE rats compared with wild-type rats, which is consistent with the loss of photoreceptors, a typical feature of RP. Although VA was significantly higher in SDxLE rats from P30 to P180, that was not the case for SC (Figure 1A,B). In SDxLE rats, a progressive SC loss was observed at P90, reaching a plateau that remained through P180, similar to P23HxLE rats at the same age (37.188 ± 1.22 vs. 37.312 ± 1.58, respectively).

Melatonin treatment increased VA in both species; it was 4% higher in SDxLE-MT rats compared with the SDxLE-vehicle group, and showed an increase of 4.4% in the P23HxLE-MT rats compared with non-treated P23HxLE rats. With regard to SC (Figure 1B), although rats treated with melatonin had higher values compared with the sham groups, no statistically significant differences were reached; in SDxLE-MT rats, the values at P180 were similar to those obtained in the SDxLE-vehicle group (41.202 ± 1.43 vs. 37.188 ± 1.22, respectively).

### 3.2. Electroretinogram Results

**Figure 2:**
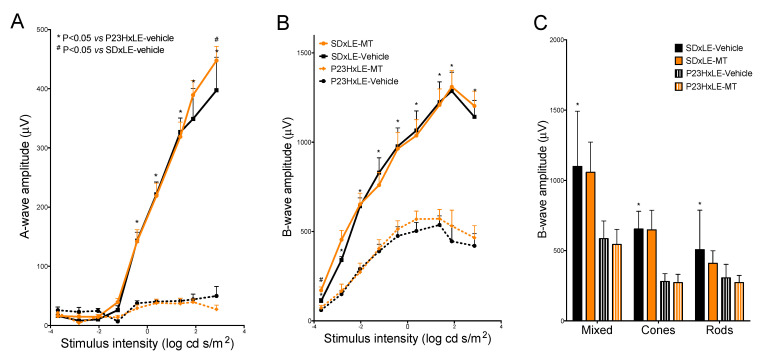
Electroretinogram recordings. Scotopic a- (**A**) and b-wave (**B**) responses for flashes of 0.0002, 0.0015, 0.0092, 0.06, 0.38, 2.38, 23.19, 78, and 722 cd.s/m^2^ intensities, and the double-flash protocol (**C**) recorded at P180 in the P23HxLE and SDxLE treatment groups: SDxLE-vehicle, SDxLE-MT, P23HxLE-MT, and P23HxLE-vehicle. Data plots show the mean ± SEM (*n* = 5). Mann–Whitney U test: # *p* < 0.05 versus SDxLE animals, * *p* < 0.05 versus P23HxLE-vehicle animals.

The scotopic a-wave was reduced in amplitude in P23HxLE rats compared with wild-type rats: in transgenic rats, the a-wave amplitude at the highest stimulus intensity (2.85 cd·s/m^2^) was only 12.5% that of wild-type rats (Figure 2A). The difference in a-wave amplitudes in P23HxLE-MT was small, with slightly lower values at 2.85 cd·s/m^2^ than the sham group. The amplitudes were significantly higher in SDxLE-MT than in the SDxLE-vehicle group.

The b-wave amplitudes were less affected than the a-waves at P180. P23HxLE rats reached 419.92 ± 67.66 µV at the highest stimulus intensity: this value amounted to only 36.8% of the corresponding value in wild-type rats (1141.24 ± 92.4 µV). The maximum amplitudes obtained for scotopic b-waves were 1.9% and 19.2% higher in wild-type SDxLE-MT and P23HxLE-MT rats, respectively, compared with the values obtained in the sham groups (Figure 2B).

Both mixed and isolated cone responses in addition to rod responses (Figure 2C) were statistically higher in wild-type SDxLE rats compared with untreated P23HxLE rats (585.32 ± 39.85 µV and 1098.98 ± 160.45 µV, respectively, in the mixed contribution, and 57.2% and 39.7% lower in the cone and rod contributions, respectively, in P23HxLE rats than in SDxLE rats). Melatonin treatment provided no benefit to P23HxLE rats or SDxLE rats with the probe flash at 1.4 log cd·s/m^2^ in the double-flash protocol.

### 3.3. Body Temperature and Locomotor Activity Rhythms

**Figure 3:**
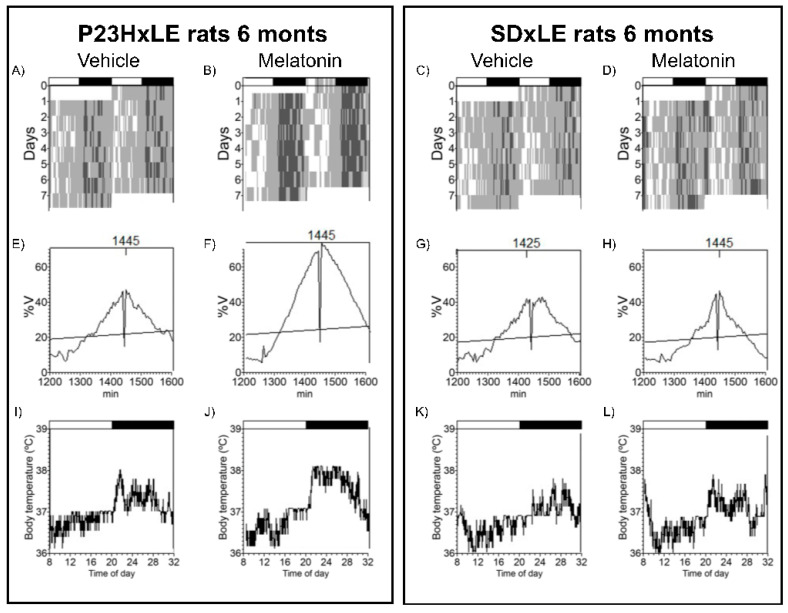
Core body temperature rhythms: representative actograms (**up**), periodograms (**middle**), and mean waveforms (**down**) obtained by analyzing P23HxLE (**left**) and SDxLE (**right**) rats without treatment, the vehicle (**left**), and treated with melatonin (**right**) at P180. The light/dark cycle is represented by dark and white horizontal bars.

**Figure 4:**
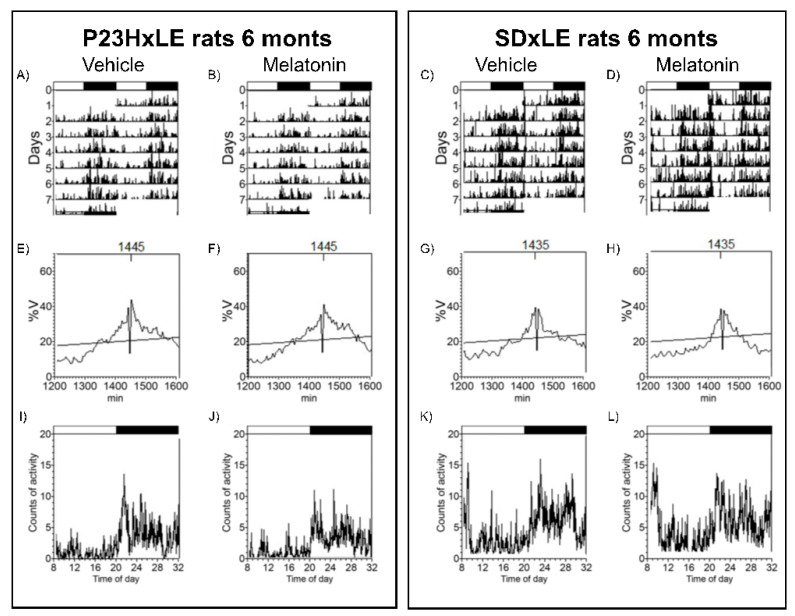
Locomotor activity rhythms: representative actograms (**up**), periodograms (**middle**), and mean waveforms (**down**) obtained analyzing P23HxLE (**left**) and SDxLE (**right**) rats without treatment, the vehicle (**left**), and treated with melatonin (**right**) at P180. The light/dark cycle is represented by dark and white horizontal bars.

Analyzed circadian parameters of body temperature and locomotor activity in P23HxLE and SDxLE rats are shown in Table 1. Animals were exposed to a 12:12 light–dark cycle and data were registered at P180 for 7 days to assess circadian rhythms with melatonin treatment. A comparative example of actograms, periodograms, and mean waveforms for P23HxLE and wild-type rats with and without treatment are shown in Figure 3 and Figure 4 (temperature and locomotor activity, respectively).

Significant differences were found between SDxLE and transgenic P23HxLE rats, highlighting the temperature–L2 parameter (the least temperature 2 h), reaching almost a displacement of 9 h when comparing P23HxLE (13:55 ± 1:24 h) to SDxLE rats (5:05 ± 2:30 h). This parameter is also significantly different in locomotor activity rhythm (P23HxLE rats: 6:45 ± 0:20 h, and SDxLE rats: 6:31 ± 0:24 h). Higher values of activity were observed in wild-type animals (4.47 ± 0.42 vs. 2.69 ± 0.28 in P23HxLE rats). The index that correlates the temperature–L2 parameter with the activity–L2 parameter is the desynchronization index L2. The synchronization between core body temperature and locomotor activity was worse in P23HxLE rats (0.60 ± 0.11) than in wild-type rats (0.19 ± 0.14). A score of 0 indicates perfect synchronization between the parameters. Treatment with melatonin decreased the desynchronization index L2 in both P23HxLE (0.26 ± 0.07) and SDxLE rats (0.09 ± 0.04), indicating a better circadian pattern in these animals than in those without treatment.

### 3.4. Oxidative Stress Parameters

**Figure 5:**
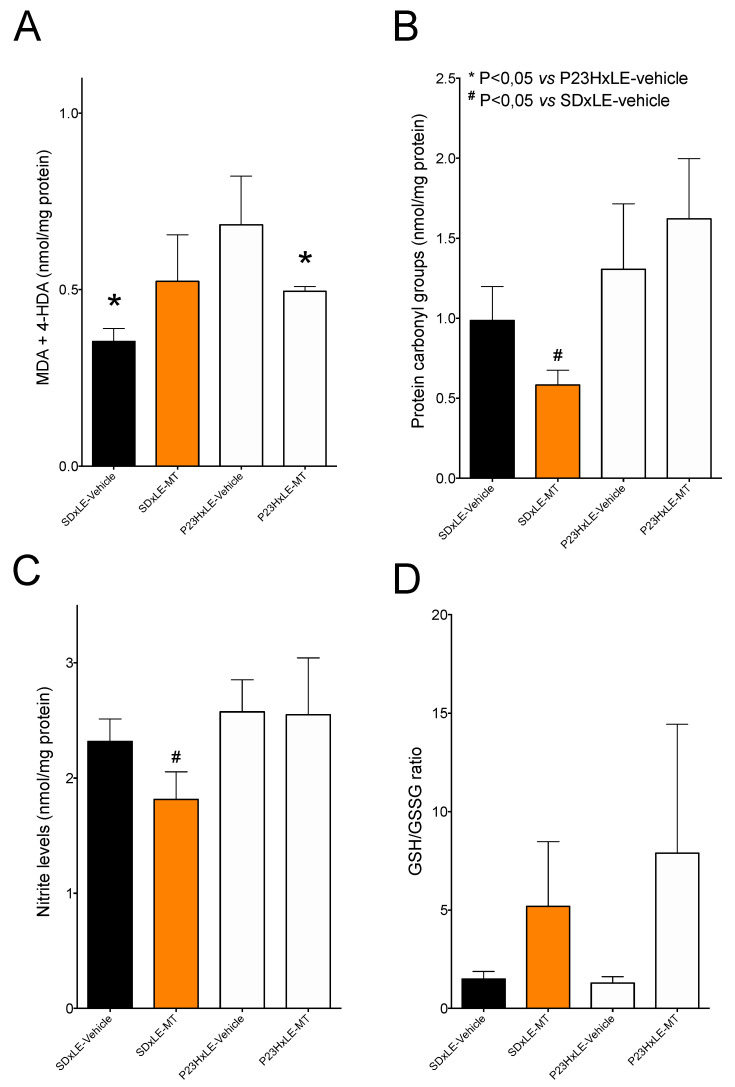
Hepatic levels of lipid peroxidation (**A**), oxidized proteins (**B**), nitrosative damage (**C**), and the GSH/GSSG ratio (**D**) in wild-type (SDxLE) rats and P23HxLE rats without treatment (vehicle) and those treated with melatonin (MT). Data represent the mean ± SEM (*n* = 5). Mann–Whitney U test: # *p* < 0.05 versus SDxLE animals, * *p* < 0.05 versus P23HxLE-vehicle animals.

P23HxLE rats showed higher levels of lipid peroxidation, measured as malondialdehyde + 4-hydroxyalkenal levels, with values of 0.684 ± 0.14 vs. 0.3536 ± 0.036 nmol/mg protein. Melatonin treatment significantly reduced these levels in transgenic P23HxLE rats, but not in wild-type SDxLE rats (Figure 5A).

Analysis of protein carbonyl group levels revealed no significant differences in oxidized proteins between SDxLE and P23HxLE animals (Figure 5B). SDxLE rats treated with melatonin showed a significant 41% reduction in oxidized proteins compared with the sham group. P23HxLE-MT rats showed no significant differences in oxidized protein levels.

Melatonin treatment showed a slight tendency to increase hepatic nitrite levels (Figure 5C), a marker of nitrosative damage, in non-treated-P23HxLE rats compared with SDxLE rats (2.58 ± 0.28 and 2.32 ± 0.19 nmol/mg protein, respectively). Similar results were obtained with melatonin treatment in the P23HxLE group (2.549 ± 0.49 nmol/mg protein); nitrite levels were significantly lower in SDxLE-MT rats compared with the sham group (1.81 ± 0.24 nmol/mg protein, a reduction of almost 22%).

No changes were detected in the GSH/GSSG ratio between groups (Figure 5D), although both groups treated with melatonin showed higher levels compared with the non-treated groups.

### 3.5. Antioxidant Defenses

**Figure 6:**
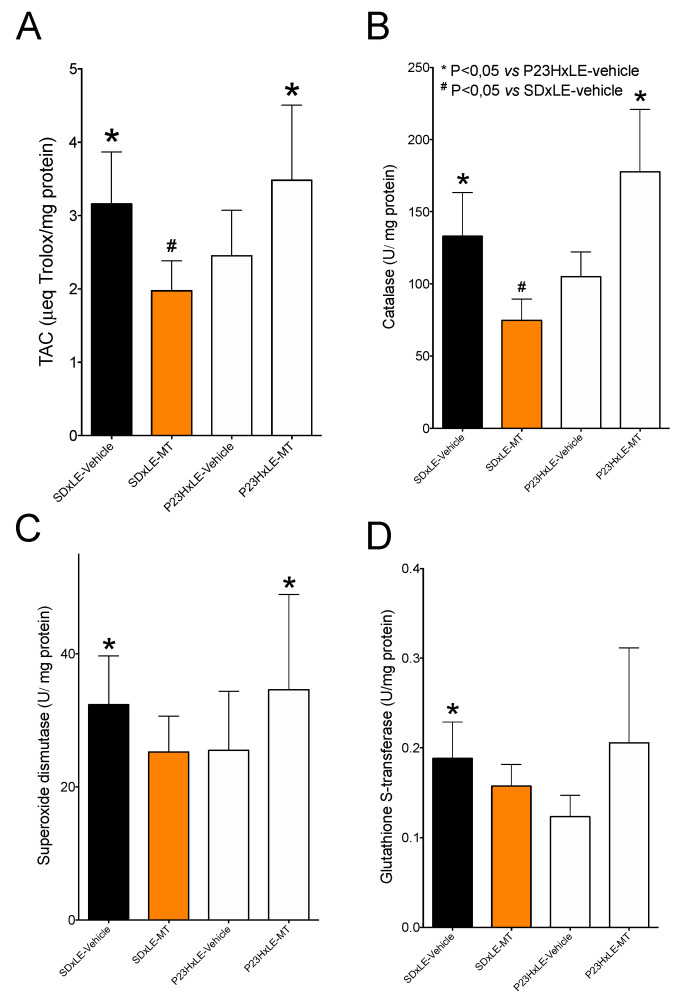
Levels of antioxidant defenses: (**A**) total antioxidant capacity (TAC), (**B**) catalase (CAT), (**C**) superoxide dismutase (SOD), and (**D**) glutathione S-transferase (GST) enzyme activities analyzed in liver tissues of wild-type (SDxLE) rats and P23HxLE rats without treatment (vehicle) and treated with melatonin (MT). Data represent the mean ± SEM (*n* = 5). Mann–Whitney U test: # *p* < 0.05 versus SDxLE animals, * *p* < 0.05 versus P23HxLE-vehicle animals.

The measurement of total antioxidant capacity (Figure 6A) showed lower values in P23HxLE rats compared with wild-type rats. Melatonin treatment increased by 42% in this parameter, but in SDxLE-MT rats the total antioxidant capacity was decreased by 37.5%. Levels of the three antioxidant enzymes (CAT, SOD, and GST) were lower in P23HxLE rats than in SDxLE rats: a reduction of 21.1% in CAT, 21.2% in SOD, and 34.4% in GST. Both CAT (Figure 6B) and SOD (Figure 6C) showed increased activity levels with melatonin treatment in P23HxLE, with CAT increasing by almost 41% and SOD increasing by 26.3%, but not GST, which did not differ significantly from the sham group.

## 4. Discussion

The present study provides data on the antioxidant and neuroprotective effects of melatonin against a retinal neurodegenerative disease, RP, in an animal model, the P23HxLE rat. The neuroprotective properties of melatonin have been demonstrated in numerous cell systems [36], animal models [37], and clinical trials [10]. Melatonin is biosynthesized not only in the pineal gland but also in other tissues, such as the retina, where it acts as a neuromodulator. In animal models of eye diseases, including glaucoma, age-related macular degeneration (AMD), cataracts, and diabetic retinopathy, melatonin has demonstrated benefits [38]. To the best of our knowledge, this is the first study to report that treatment with melatonin significantly improves VA in an animal model of RP with slow degeneration of the retina. Its potential as a therapeutic agent for aging-related eye diseases has been probed in wild-type (SDxLE) animals, which showed greater VA and modest improvement in CS and electroretinogram results. In a previous study [39], melatonin treatment delayed the progression of AMD in human patients, with no deterioration of VA. The photoreceptor loss and subsequent degeneration of visual function, assessed by both optometry and electroretinography, has been widely demonstrated in P23HxLE rats [40,41]. At this stage of the disease, improvements are of short duration, with retinal preservation for up to approximately 6 months [42,43,44]. Numerous studies have reported the involvement of melatonin in retinal physiology, regulating some functions such as dopamine release, disk shedding, light sensitivity, and intraocular pressure among others that are dependent on retinal circadian rhythms [45]. Different findings regarding the effects of melatonin on electroretinograms have been reported. On the one hand, oral administration of melatonin during the day decreased a- and b-wave amplitudes, especially affecting the cone response: this seems to be correlated with a decrease in dopamine levels, which enhances horizontal cell coupling, and has an inhibitory effect on cones [46]. On the other hand, Baba et al. [47] reported higher a- and b-wave amplitudes in mice treated with an intraperitoneal injection of melatonin during the day: differences in these results could be due to the nocturnal and diurnal characteristics of the animal models studied, with demonstrated differences relating to hormonal and neurotransmitter rhythms [48]. Previous studies in albino P23H rats showed that the maximum b-wave recorded at 14 months of age was significantly higher in melatonin-treated P23H rats than in control animals [26]. Our study, in addition to assessing the effects of melatonin on ERGs, also wanted to assess its effects on VA. Therefore, to avoid the effects of albinism in the measurement of VA and to use the model to resemble the clinic, where most patients have heterozygous RP, in our study the RP model was designed with pigmented animals with heterozygous P23H (P23HxLE). In our study, the administration of melatonin to the P23HxLE rat improved parameters of visual function: VA, CS, and retinal electrical activity, indicating better cell preservation and delayed progression of the disease. Melatonin supplementation in wild-type rats also provided improvements in every visual function parameter analyzed: VA, CS, and retinal electrical activity, suggesting that these antioxidant treatments could be useful for patients with age-related ocular diseases, such as AMD.

The retina is involved not only in vision but also in other non-visual functions, such as the regulation of circadian rhythms. Light is detected by a subset of retinal ganglion cells characterized by a special opsin, melanopsin. These cells send ambient light information to a specific region of the central nervous system, the suprachiasmatic nucleus, which is the main circadian pacemaker. This nucleus is responsible for regulating the 24 h day/night cycle and other functions, such as the secretion of melatonin from the pineal gland. In the absence of light in the retina, the circadian system loses this synchronization with the environment and acquires its own rhythm, making it difficult to differentiate the 24 h environmental day/night cycle [49]. This is a common feature in blind people, especially those who have lost light perception. This abnormal 24 h period causes sleep disturbances and free-running circadian rhythms, which affect parameters such as core body temperature, activity, and melatonin concentration, among others [50,51]. In the present study, P23HxLE rats showed a delayed entrainment rhythm based on the analysis of the L2 parameter in temperature, with a displacement of almost 9 h in these animals, and the desynchronization index L2, with a difference of 7 h between the lowest 2 h of temperature and activity. There was no significant difference, however, in non-parametric variables such as relative amplitude, intradaily variability, and interdaily stability. These findings are supported by the results of another study [51] that demonstrated that the retinal melanopsin system is impaired and circadian rhythms are disturbed in late-stage RP. Melatonin administration to wild-type animals significantly reduced the desynchronization index L2, similar to in P23HxLE rats, demonstrating the ability of this molecule to synchronize 24 h endogenous rhythms, as in physiological conditions [52]. Lax et al. [26] supplied melatonin to P23H-3 rats at a concentration of 2 mg/kg/day for 16 months, and showed reduced retinal degeneration as well as reduced circadian rhythmicity impairment, supporting our results.

The key role that oxidative stress plays in eye diseases is widely known: free radicals react with different biomolecules, such as lipid membranes, proteins, and DNA, thereby triggering cellular damage and inducing inflammation and apoptosis [53]. In a previous study [4], we demonstrated that P23HxLE rats have higher levels of various hepatic oxidative stress parameters, such as lipid peroxidation and nitrite levels, and a higher GSH/GSSG ratio with age. Wild-type animals showed lower levels of lipid peroxidation than P23HxLE rats, but we found no significant differences in oxidized proteins, nitrosative damage, or the GSH/GSSG ratio between them. Melatonin, due to its high antioxidant properties, acts directly as a free radical scavenger, and indirectly enhances antioxidant enzymes, such as CAT, SOD, glutathione reductase, and glutathione peroxidase. Melatonin also inhibits some pro-oxidant enzymes and has thus been used in numerous studies to reduce levels of peroxidized lipids in different diseases [54,55,56]. Our results showed that, in P23HxLE rats, melatonin significantly reduced levels of lipid peroxidation compared with untreated P23HxLE rats. This was not the case in wild-type rats; no significant differences in lipid peroxidation levels were detected following melatonin treatment.

Regarding the other oxidative stress markers analyzed, P23HxLE rats tended to have increased protein carbonyl group and nitrite levels and a slightly lower GSH/GSSG ratio, indicating impaired cell health compared with wild-type rats. Although some studies that evaluated the beneficial effects of melatonin administration on different diseases demonstrated decreased levels of nitrosative damage, which affects the expression of oxide nitric synthase and reduces protein oxidative damage [57,58,59,60,61], we observed no significant changes in nitrosative damage in the melatonin-treated P23HxLE rats in the present study. Wild-type rats treated with melatonin, however, showed lower levels of these parameters, indicating a possible positive effect of melatonin as a supplement for aging, considering that there is a physiological decrease in melatonin secretion and that some age-related diseases are associated with it [62]. Melatonin enhances glutathione peroxidase activity, and therefore increases the GSH/GSSH ratio due to a higher GSH concentration [63,64], similarly to our results in P23HxLE and wild-type rats, which showed higher values in this parameter compared with the corresponding sham groups.

Counteracting excess reactive oxygen species is the antioxidant system, comprising both non-enzymatic and enzymatic molecules, which acts to maintain cellular homeostasis. An imbalance between reactive oxygen species and antioxidant defenses (known as oxidative stress) is associated with aging (and age-related diseases) and neurodegenerative diseases, such as RP [65,66]. Consistent with previous findings [4], measurements of total antioxidant capacity and the three main antioxidant enzymes (i.e., SOD, CAT, and GST) revealed impaired antioxidant defenses in P23HxLE rats. In addition to increased production of free radicals (reactive oxygen/nitrogen species), lower levels of antioxidants are associated with many neurodegenerative diseases; however, whether this is a consequence of the progression of disease or a primary cause remains unanswered [67]. The ability of melatonin to protect cells against oxidative damage has been discussed recently. Moniruzzaman et al. [68] demonstrated that melatonin ameliorates H_2_O_2_-stressed hepatocytes, leading to higher levels of CAT and SOD compared to those without melatonin supplementation. In a clinical trial with patients who suffer from carotid artery stenosis [69], melatonin administration (6 mg/day, 3 days before and after surgery) increased the expression levels of CAT and SOD compared with a placebo group. Our results, in which P23HxLE-MT rats showed higher levels of CAT and SOD activity, are consistent with these previous reports. Some antioxidant enzymes are regulated by the Keap1-ARE (Keap1 antioxidant response element) pathway, which is dependent on Nrf2, a transcription factor activated in oxidative stress situations that plays a key role in the central nervous system and neurodegenerative diseases [70]. One of these enzymes is GST: P23HxLE rats supplemented with melatonin exhibit higher values of GST activity compared with the sham group. These results are consistent with those of other studies, showing that melatonin upregulates this antioxidant enzyme and others depending on this transcriptional factor, suppressing its degradation and enhancing its nuclear translocation [71,72].

In this work, melatonin treatment markedly reduced total antioxidant capacity and CAT activity in wild-type rats and slightly decreased SOD and GST activity. Melatonin is one of the safest biological molecules known, with no adverse effects detected in studies that evaluated concentrations of up to 800 mg/kg [73]. Additional studies are needed, however, to determine the secondary effects of melatonin, because very few studies have evaluated the effects of long-term melatonin supplementation [74,75].

## 5. Conclusions

In this study, treatment with melatonin improved every visual function parameter analyzed: visual acuity, contrast sensitivity, and retinal electrical activity in P23HxLE rats, an RP model, indicating better cell preservation and delaying the progression of the disease. Melatonin additionally improved circadian synchronization in P23HxLE rats, which is compatible with its known synchronizing function of biological rhythms. Melatonin furthermore reduced oxidative stress in P23HxLE rats, probably due to its antioxidant and anti-inflammatory properties. Clinical trials are needed to evaluate the effect of melatonin in patients with initial RP who may benefit from a slowdown in the progression of the disease by reducing oxidative stress and improving visual function. Patients with an advanced stage of RP may benefit from better circadian synchronization despite their retinal degeneration and loss of visual function.

In addition, in this study wild-type animals up to 180 days old also showed better visual function, circadian synchronization, and oxidative status with melatonin treatment, indicating a possible protective effect against age-related damage. Further studies are needed to determine if the long-term administration of melatonin in healthy organisms could be useful in age-related ocular diseases.

## Figures and Tables

**Table 1:** Circadian parameters.

	SDxLE				P23HxLE			
	Temperature		Locomotor Activity		Temperature		Locomotor Activity	
	Vehicle (*n* = 5)	MT (*n* = 5)	Vehicle (*n* = 5)	MT (*n* = 5)	Vehicle (*n* = 5)	MT (*n* = 5)	Vehicle (*n* = 5)	MT (*n* = 5)
Rhythm Parameters								
Mesor (°C)	36.96 ± 0.04	36.93 ± 0.06	4.47 ± 0.42 *	4.45 ± 0.24	37.16 ± 0.13	37.00 ± 0.06	2.69 ± 0.28	3.09 ± 0.48
Amplitude (°C)	0.37 ± 0.01 *	0.46 ± 0.03 #	2.67 ± 0.35	2.44 ± 0.17	0.51 ± 0.06	0.57 ± 0.13	1.90 ± 0.17	1.70 ± 0.17
Acrophase (min)	1118.26 ± 23.66	1077.83 ± 15.82	1145.71 ± 35.07	1191.68 ± 27.01	1022.90 ± 30.69	1018.58 ± 4.63	1056.88 ± 21.28	980.50 ± 43.69
Acrophase (h:min)	2:43 ± 0:23	2:02 ± 0:15	3:10 ± 0:35	3:56 ± 0:27	1:07 ± 0:30	1:03 ± 0:04	1:41 ± 0:21	0:25 ± 0:43
Variance (%)	26.08 ± 1.076 *	31.05 ± 4.165	11.77 ± 1.59	10.56 ± 1.98	38.03 ± 4.806	40.65 ± 9.102	13.24 ± 1.39	9.08 ± 2.75
Period (min)	1445.00 ± 20.00	1440.00 ± 5.00	1441.67 ± 3.33	1437.50 ± 2.50	1442.50 ± 2.50	1442.50 ± 2.50	1442.50 ± 2.50	1457.50 ± 13.15
Non-Parametric Variables								
IS	0.63 ± 0.03	0.63 ± 0.01	0.33 ± 0.02	0.34 ± 0.02	0.69 ± 0.04	0.69 ± 0.03	0.32 ± 0.03	0.26 ± 0.03
IV	0.25 ± 0.03	0.24 ± 0.03	0.96 ± 0.01	0.95 ± 0.03	0.18 ± 0.03	0.20 ± 0.04	1.08 ± 0.07	1.07 ± 0.01
RA	0.01 ± 0.00	0.01 ± 0.00	0.50 ± 0.02	0.55 ± 0.03	0.01 ± 0.00	0.01 ± 0.00	0.60 ± 0.04	0.49 ± 0.07
L2 (hh:mm)	5:05 ± 2:30 *	6:17 ± 0:32	6:31 ± 0:24 *	5:12 ± 0:30	13:55 ± 1:24	10:02 ± 0:11	6:45 ± 0:20	6:55 ± 0:43
VL2 (°C)	37.53 ± 0.08	37.55 ± 0.08	4.67 ± 0.89 *	4.54 ± 0.32	37.43 ± 0.07	37.30 ± 0.11	2.82 ± 0.13	2.74 ± 0.30
Media (°C)	37.43 ± 0.04	37.44 ± 0.04	4.87 ± 0.43 *	4.85 ± 0.25	37.48 ± 0.03	37.48 ± 0.05	3.07 ± 0.29	3.48 ± 0.49
CFI	0.30 ± 0.00	0.29 ± 0.00	0.60 ± 0.01	0.61 ± 0.01	0.29 ± 0.01	0.30 ± 0.00	0.66 ± 0.03	0.61 ± 0.03
DesynchroIndex L2	-	-	0.19 ± 0.14	0.09 ± 0.04 #	--	-	0.60 ± 0.11	0.26 ± 0.07 *

Circadian parameters recorded from P23HxLE and SDxLE rats after 6 months of treatment. Mesor: mean of 24 h time series. Amplitude: one-half the peak-to-trough difference of the 24 h rhythm. Acrophase: peak time relative to maximum value. IS: interdaily stability. IV: intradaily variability. MT: melatonin. RA: relative amplitude. L2: the least temperature 2 h. CFI: circadian function index. DesynchroIndex L2: desynchronization index L2. # *p* < 0.05 versus SDxLE-vehicle, * *p* < 0.05 versus P23HxLE-vehicle.

## Data Availability

The data presented in this study are available in this manuscript.
